# Mepyramine targets mutant Nav1.7 channels to relieve pain and erythema in primary erythromelalgia patients

**DOI:** 10.3389/fmed.2025.1744968

**Published:** 2025-12-12

**Authors:** Myriam Ducrocq, Virginie Penalba, Laura Castillo, Christine Bodemer, Céline Greco, Patrick Delmas

**Affiliations:** 1 Centre de Recherche en CardioVasculaire et Nutrition, Aix-Marseille Univ, INSERM, INRAE, Marseille, France; 2 CNRS EMR7005, Ion Channels and Interoception, Marseille, France; 3 Department of Pediatric Dermatology, Assistance Publique Hôpitaux de Paris (APHP), Necker-Enfants Malades Hospital, Paris Cité University, Paris, France; 4 Department of Pain and Palliative Medicine Unit, Assistance Publique Hôpitaux de Paris (APHP), Necker-Enfants Malades Hospital, U1163 Imagine Institut, Paris Cité University, Paris, France

**Keywords:** erythromelalgia, pain, Nav1.7, sodium channels, erythema, antalgic, mepyramine

## Abstract

Gain-of-function mutations in *SCN9A*, which encodes the Nav1.7 voltage-gated sodium channel, are known to cause primary erythromelalgia (PEM). This condition is characterized by recurrent episodes of erythema, burning pain, and warmth in the extremities. These genetic insights have spurred the development of Nav1.7 blockers as a promising therapeutic strategy for PEM. However, translating these findings into effective clinical treatments has remained challenging. In this study, we demonstrate that mepyramine, a compound previously shown to alleviate pain in animal models, effectively targets hNav1.7 channels carrying PEM-associated gain-of-function mutations, providing substantial pain relief in PEM patients. Using voltage-clamp recordings in human embryonic kidney (HEK) 293 cells, we demonstrated that mepyramine inhibits hNav1.7 channels carrying three distinct PEM mutations, I848T, L858F, and L1267V, which differentially affect the gating properties of hNav1.7. Importantly, mepyramine’s efficacy was consistent regardless of how these mutations altered channel activation or inactivation properties. To evaluate its clinical potential, we administered a high-dose topical formulation of mepyramine to a group of PEM patients suffering from severe pain that was unresponsive to conventional analgesics, including cases with identified *SCN9A* mutations. This treatment rapidly and durably reduced burning pain and erythema, providing meaningful relief for patients who had not responded to, or could not tolerate, previous therapies. These results suggest that mepyramine can inhibit PEM-associated Nav1.7 channel mutants and may offer a new therapeutic approach for PEM patients.

## Introduction

Primary erythromelalgia (PEM) is a rare disorder characterized by intermittent episodes of severe, localized burning pain accompanied by intense redness and warmth of the skin in affected extremities (1–3). PEM typically affects the soles of the feet and the palms of the hands symmetrically, and sometimes parts of the face. PEM attacks can be triggered by hot weather, fever, exercise, or wearing socks or tight shoes. These episodes can occur infrequently, multiple times a day, or even persist constantly as the disease progresses, leading to significant disability (4). Patients often soak their feet in ice water during these attacks to relieve the pain (5, 6).

PEM may present as a primary disorder, arise from pathogenic *SCN9A* variants as genetic PEM, or remain idiopathic when no underlying cause is identified, and it can occur either sporadically or as an autosomal-dominant inherited condition. In contrast, secondary erythromelalgia (EM) is associated with underlying conditions such as diabetes mellitus, vascular disease, hypertension, peripheral neuropathies, collagenopathies, hematologic disorders, myeloproliferative diseases, and drug-induced reactions, including cases triggered by medications or dietary supplements, which underscores the importance of obtaining a thorough pharmacologic and dietary history (4, 7, 8). The incidence of PEM or secondary EM is estimated to be 0.4–2 cases per 100,000 people per year in the USA and Europe, with a female-to-male ratio of approximately 2–3:1 (1, 2, 4, 9, 10).

Genetic research has identified a link between cases of PEM and mutations in the Nav1.7 sodium channel *α*-subunit, which is encoded by the *SCN9A* gene (11–14). The Nav1.7 sodium channel is predominantly expressed in nociceptive dorsal root ganglia (DRG) as well as in sympathetic and enteric ganglia (15–17). Like many Nav channels, Nav1.7 enables depolarizing stimuli to trigger action potential (AP) generation and propagation in neurons (18, 19). Nav1.7 also serves as a “threshold gate” in AP generation, as it can amplify small, slow depolarizations, bringing the membrane potential closer to the AP threshold (20). *SCN9A* mutations have been associated with various pain disorders, including gain-of-function paroxysmal extreme pain disorder (PEPD) and the loss-of-function disorder congenital insensitivity to pain (CIP) (21–23).

To date, about 25 mutations in the *SCN9A* gene have been identified as responsible for PEM (21). Most of these mutations are localized within the S4 segment, S4/S5 linker, or the S5 and S6 segments, which collectively influence the voltage dependence of activation and inactivation in Nav1.7 channels (21, 22). Functional studies have shown that PEM mutations increase sensory neuron excitability through hyperpolarizing shifts in activation (where mutant channels open more readily at lower voltages) or depolarizing shifts in steady-state inactivation (where mutant channels are less likely to inactivate at higher voltages) (21).

PEM is generally resistant to most pharmacologic therapies. Genetic research has supported efforts to develop Nav1.7 blockers as a potential therapeutic strategy. In recent decades, lidocaine, mexiletine, and carbamazepine have been used for Nav-related pain disorders (24–27). Carbamazepine, in particular, has been shown to partially normalize abnormal activation gating and reduce hyperexcitability in sensory neurons carrying gain-of-function Nav1.7 and Nav1.8 mutations linked to painful peripheral neuropathies and erythromelalgia, in line with the clinical responsiveness observed in affected individuals (28–31). All these drugs act as nonselective Nav channel blockers, thereby inhibiting the sodium influx required for action-potential generation. However, their clinical utility is constrained by relatively narrow therapeutic windows and significant off-target effects on cardiac, cognitive, and motor functions (32). Additionally, some individuals show therapeutic resistance, which may be explained by genetic polymorphisms in Nav1.7 mutations.

As a result, neither Nav channel blockers nor other pharmacologic agents, such as antidepressants and anticonvulsants (33), have shown complete efficacy, leading to the need for polypharmacy in most patients. Consequently, PEM treatment remains a stepwise, trial-and-error approach, with no single therapy found to be universally effective.

Our recent research has shown that mepyramine, a first-generation antihistamine that targets the histamine H1 receptor (H1R) (34), also has analgesic effects through direct action on pain-transmitting sodium channels in mice (35). Mepyramine acts as an effective inhibitor of Nav isoforms, including Nav1.7 in nociceptors (35), where this channel contributes to nociception and pain-related disorders (36). Additionally, mepyramine inhibits Nav1.8 and Nav1.9, two key sodium channel isoforms involved in nociception and the development of pathological pain (35, 37, 38). Local administration of mepyramine has been shown to reduce nociceptive signal transmission in skin-nerve preparations and provides effective analgesia in various mouse models of inflammatory and neuropathic pain (35). Together, these findings support the potential of mepyramine as a topical analgesic agent.

We demonstrate here that mepyramine effectively antagonizes the abnormal activity of hNav1.7 mutant channels containing the missense PEM mutations I848T, L858F, and L1267V. Mepyramine successfully inhibits the activity of these hNav1.7 mutant channels, regardless of the underlying mechanisms driving their gain-of-function. Given that PEM symptoms appear localized, we employed a topical approach to minimize systemic effects of the medication. We developed a mepyramine cream for administration to PEM patients, including those with identified *SCN9A* mutations, with the goal of directly targeting both the underlying etiology and associated pain symptoms. Topical application of mepyramine significantly improved vascular and neurophysiological symptoms in PEM patients. Collectively, these findings provide strong evidence that mepyramine holds promise as a topical analgesic and anti-inflammatory agent in both preclinical and clinical settings for PEM.

## Materials and methods

### Plasmids, DNA cloning, and mutagenesis

For expression in mammalian cells, hNav1.7 L1267V (c.3799C>G, p,leu1267Val), I848T (c.2543T>C) and L858F (c.2572C>T, p.leu858Phe) mutants were generated based on WT human Nav1.7 (*SCN9A* transcript variant 1 containing 1977 amino acids, NM_002977.3) in a modified pCDNA3_IRES-AcGFP1 vector (FLGREEN) (23) by commercial site-directed mutagenesis (Genewiz, Germany). Constructs were verified by commercial DNA sequencing (Genewiz, Germany).

### Expression of hNav1.7 mutants with hβ1/β2 Na^+^ subunits in HEK293T cells

Human embryonic kidney (HEK) 293T cells were cultured in Dulbecco’s modified Eagle’s medium containing 10% heat-inactivated FBS and 1% penicillin/streptomycin. Cells were plated onto 12-mm round glass poly-D-lysine-coated coverslips placed in 24-well plates and transfected using lipofectamine 2000 (Invitrogen) using 1.5 μg/mL of hNav1.7 cDNAs (WT, I848T, L858F or L1267V) and 1.5 μg/mL of hβ1 and hβ2 Nav subunits (JC5 plasmids) (23). Patch clamp experiments were conducted on green fluorescent cells 48 h post-transfection.

### Patch-clamp recordings of hNav1.7 currents in HEK293T cells

Whole-cell voltage-clamp recordings were performed on isolated HEK293T cells expressing WT or mutant Nav1.7 channels at room temperature. Recordings were made using borosilicate electrodes (Harvard Apparatus, Holliston, Massachusetts, United States) with a resistance of 3–4 MΩ when filled with an intracellular solution consisting of (in mM): 130 CsCl, 10 Hepes, 8 NaCl, 0.4 NaGTP, 4 MgATP, 1 MgCl_2_, 1 CaCl_2_ and 10 EGTA (adjusted to pH 7.3 with CsOH, ~300 mOsm/L). The bath solution consisted of (in mM): 140 NaCl, 3 KCl, 1 MgCl_2_, 1 CaCl_2_, 10 HEPES and 10 glucose (pH 7.3 with NaOH, adjusted to 300–305 mOsm). Sodium currents were leak-subtracted using a P/6 protocol and voltage errors were minimized using 75–80% series resistance compensation. Recordings were using an Axopatch 200B amplifier (Axon Instruments, Boston, Massachusetts, United States), filtered at 1–2 kHz, and digitally sampled at 5–20 kHz. Recordings began following a 5-min equilibration period after establishing the whole-cell configuration to allow Cs^+^ to equilibrate and Na^+^ channels to recover from inactivation present at rest.

To measure activation, cells were stepped from the holding potential of −80 mV to potentials ranging from −60 to +60 mV in 5 mV increment for 25 ms. Peak Nav1.7 currents obtained from activation protocols were converted to conductance values using the equation,


$$G=I/(V_{m}-E\mathrm{Na})$$

where *G* is the macroscopic sodium conductance, *I* is the peak inward current, *V*_m_ is the membrane potential used to elicit the current, and *E*Na is the reversal potential for sodium ions. *E*Na was extrapolated for each recording by linear fitting of the current–voltage relationship and determining the intersection with the voltage axis. Normalized availability data were fitted using a standard single-phase Boltzmann distribution of the form,


$$\frac{G}{G_{\max}}=1/(1+\exp[\frac{\left(V_{m}-V_{1/2}\right)}{k}])$$

where *V*_1/2_ is the midpoint of activation and k is the slope factor.

To probe the effects of mutations and mepyramine on inactivation, availability of noninactivated channels was assessed using a series of 20 ms, 200 ms or 5 s depolarization pre-pulses. For fast inactivation, pre-pulses ranged from −95 to 0 mV from a holding potential of −100 mV and were immediately followed by a 25 ms depolarization to 0 mV. To assess slow inactivation, the 5-s depolarization pre-pulses ranged from −105 to 0 mV from a holding potential of −110 mV and were followed by a 20-millisecond hyperpolarization pulse to −120 mV to facilitate channel recovery from fast inactivation. The normalized peak inward current amplitude for each test pulse is displayed as a function of the pre-pulse potential and is fitted to the standard single-phase Boltzmann equation for both fast and slow inactivation,


$$\frac{I}{I_{\max}}=1/(1+\exp[\frac{\left(V_{m}-V_{1/2}\right)}{k}])$$

where *V*_1/2_ is the midpoint of fast or slow inactivation and *k* is the slope factor.

### Mepyramine *in vitro* treatment

Mepyramine salt (Sigma-Aldrich) was dissolved in extracellular solution to create a 100 mM stock solution, with the pH adjusted to 7.4. Subsequent dilutions were prepared in a standard external Krebs solution to achieve the desired concentrations. The addition of mepyramine did not affect the osmolarity of the extracellular solution. Cells were superfused at a laminar flow rate of 2 mL/min using a gravity-driven perfusion system with polyethylene tubing. Mepyramine was applied for at least 2 min to reach the inhibitory plateau effect before washout.

### Patient enrollment, case series, and genomic analysis

#### Participants

Our study is a retrospective analysis of an open-label case series, conducted without randomization or a control group, utilizing anonymized data from PEM patients treated during routine clinical practice at Necker-Enfants Malades Hospital [Assistance Publique-Hôpitaux de Paris (AP-HP), Paris, France] between January 2, 2022, and July 30, 2024. The study population consisted of patients diagnosed with PEM who experienced severe pain unresponsive to conventional analgesics. Symptoms began in early childhood for most individuals, with some developing erythromelalgia symptoms only at the onset of adolescence. These patients were managed using a compounded preparation containing mepyramine.

The institutional review board of the reference center for genetic skin diseases-Necker-Hospital (Paris, France), approved the study. The study was performed in accordance with the Declaration of Helsinki and its subsequent amendments, good clinical practice guidelines (CHMP/ICH/135/1995 and integrated addendum), and the general data protection regulation (GDPR, Regulation (EU) No. 2016/679 and local regulations). Written informed consent was obtained from the patients, or their legal guardians for children (<18 years old), for the publication of their data included in this article.

Patients with refractory PEM pain were referred to the joint pain and dermatologic consultations if they had persistent neuropathic pain defined as an numerical rating scale (NRS) pain score of 4 or more for more than 3 months, despite prior treatment with conventional analgesics [e.g., paracetamol, non-steroidal anti-inflammatory drugs (NSAIDs), opioids, antidepressants or antiepileptics] or poor tolerance to these prior treatments. Whole exome sequencing was performed at the molecular genetics laboratory (Henri Mondor Hospital, France), and variants detected in the *SCN9A* gene were confirmed by Sanger sequencing. Rare variants with an allele frequency of <1% in the Exome Variant Server were selected for further analysis.

Mepyramine cream, an oil-in-water emulsion containing 20% mepyramine, was prepared at Pharmacy Delpech (Paris), which specializes in magistral preparations. No commercial formulation of 20% mepyramine is currently available, and this compounded preparation has been used exclusively within our institution. Patients were instructed to apply the mepyramine cream to the affected areas of the feet and/or hands during episodes of pain. No limitations were placed on the frequency of application to regions experiencing burning pain. The selection of the 20% concentration was based on preliminary data indicating that lower concentrations (2–5%) were ineffective and failed to provide adequate analgesia in patients with PEM. Patients were advised to apply a thin, uniform layer of the cream, rub it gently, and, when treating painful areas of the hands, to avoid washing their hands for at least 30 min after application to allow optimal absorption. There was no need for foot washing after application of the cream. No other new analgesics or other pain-relieving nonpharmacologic therapies were to be used during the study period. Patients were also informed of the risk of skin irritation at application sites, and of safety issues regarding the need to avoid ingesting the cream and bringing it in contact with the eyes. Patients with pain intensity lower than 4 on the Numeric Pain Rating Scale (NPRS) were excluded. Adult patients with open lesions on the hands or feet, or those with dementia or unable to apply the cream independently, were also excluded.

### Patient follow-up

A regular follow-up of patients with PEM is arranged as part of the care provided. Pain intensity, frequency, duration of crisis and the occurrence of adverse effects (AEs), were evaluated by the physician during teleconsultations on day D7, D15, D21, and during face-to-face consultations at months M1 and M3. These visits were part of the routine follow-up of the patients. Patients were asked to keep a record of their pain and consumption of other analgesics in a diary, which was then evaluated at the monthly visits.

### Assessment criteria

We have evaluated as a primary assessment criterion the effectiveness of mepyramine cream for relieving pain after 3 months of treatment, and as secondary assessment criteria the safety of mepyramine cream and its effectiveness for reducing the frequency and the duration of PEM crisis. Pain intensity was measured on a NPRS ranging from 0 (“no pain”) to 10 (“worst pain”) both before and after the application of the cream. The intensity, duration, and frequency of pain attacks were assessed by the patients and reported to the doctors over a 3-month period. The Minimally Clinically Important Difference (MCID) was the smallest improvement in a symptom (e.g., pain intensity) that a patient perceives as beneficial. MCID was evaluated using the percentage reduction from baseline on the numerical rating scale. The categorization of “minimal” (≥10–30%), “moderate” (≥30–50%), and “much” (≥50%) relief is based on validated cut-offs derived from patient-centered outcomes research in chronic and neuropathic pain (39).

### Adverse events

Patients underwent monthly laboratory monitoring (complete blood count, serum electrolytes, liver and renal function tests) and systematic surveillance for known mepyramine-related side effects through monthly in-person visits and telephone follow-up, assessing autonomic, neurological, and hypersensitivity reactions. Adverse effects were recorded using the standard hospital system, based on Medical Dictionary for Regulatory Activities (MedDRA) terminology, and graded as severe (serious or life-threatening), moderate (non-serious but requiring treatment discontinuation), or mild (non-serious allowing treatment continuation).

### Statistics

Data are presented as numbers and percentages or means ± the error of the mean (S.E.M.). Depending on the sample size and pairing, statistical analyses were performed using unpaired or paired two-tailed *t*-tests, one-way ANOVA, Tukey’s or Sidak’s multiple comparisons test and the Wilcoxon test as appropriate. Given the small sample sizes (*n* < 7) in some experiments, the statistical analyses are intended to be exploratory and provide preliminary insights that should be interpreted with appropriate caution. Differences were considered statistically significant if *p* < 0.05.

### Mepyramine inhibits the domain II PEM hNav1.7 mutants I848T and L858F

**Figure 1:**
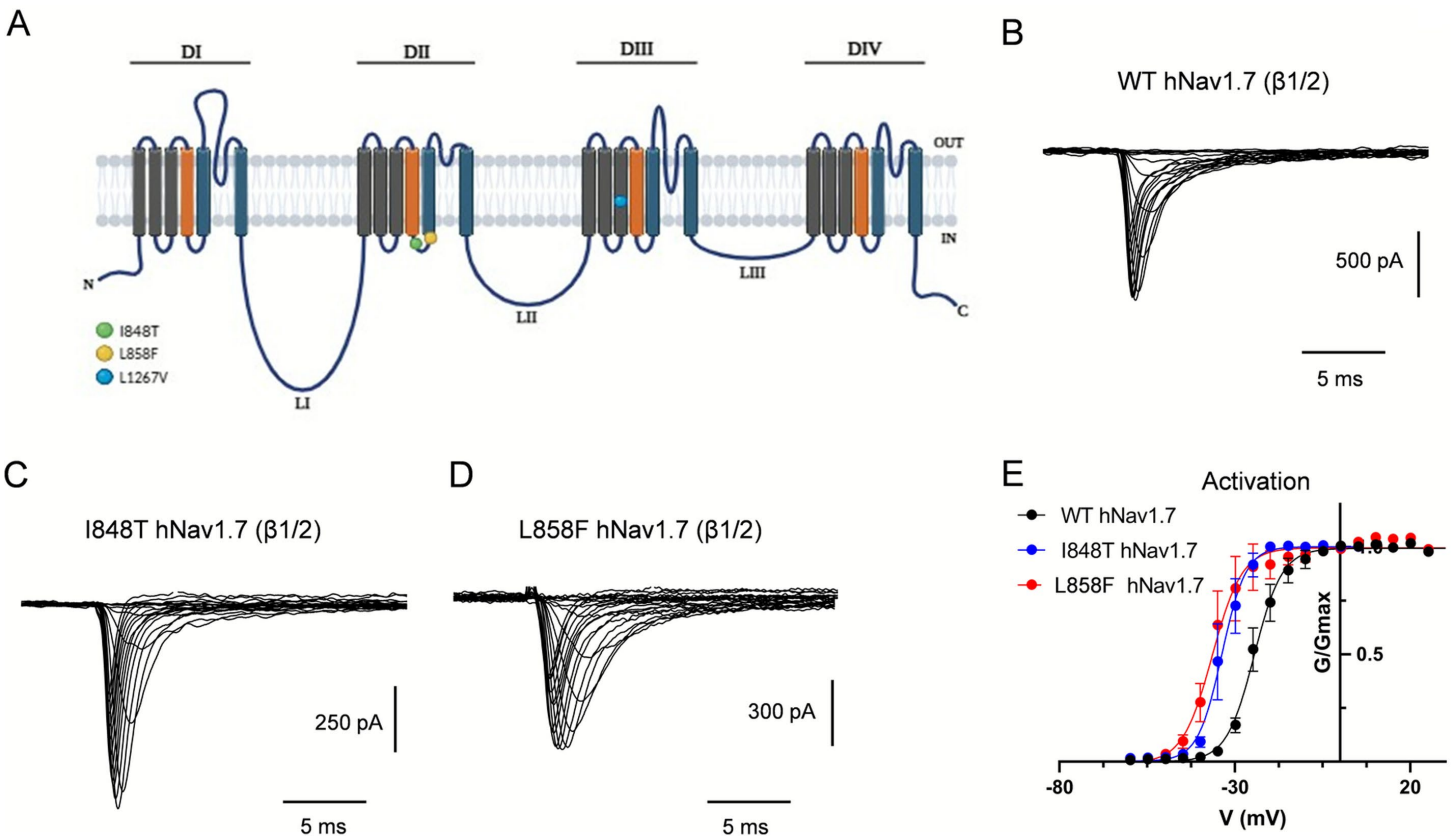
The I848T and L858F mutations modify the voltage-dependent activation of hNav1.7. **(A)** Diagrammatic scheme of mutated regions within hNav1.7. Schematic representation of hNav1.7 with the location of the I848T, L858F and L1267V mutations, which lead to PEM. Mutations are indicated with colored circles. The *α*-subunit of hNav1.7 consists of four homologous domains (DI–DIV), each of which consists of six transmembrane segments. Within each domain, S4 represents the voltage-sensing domain (depicted in orange) and S5–S6 and their extracellular linker comprise the pore module (depicted in blue). Three intracellular loops (L1–L3) connect the four domains. The inactivation particle has been identified as a conserved IFMT motif in the cytoplasmic region linking DIII and DIV. **(B–D)** Current traces recorded from HEK293 cells co-expressing either wild-type hNav1.7 **(B)**, I848T **(C)** or L858F **(D)** mutant channels, with β1- and β2-subunits. Currents were elicited with 25 ms test pulses to potentials ranging from −60 to 60 mV. For the sake of clarity not all traces are shown. **(E)** I848T and L858F mutant channels display a hyperpolarized voltage dependence of activation compared to wild-type hNav1.7. Error bars indicate mean ± SEM. *V*_1/2_ ± SEM (mV): WT hNav1.7, −21.18 ± 0.31; I848T, −34.61 ± 0.45; L858F, −36.59 ± 0.32.

Individual PEM mutations and their locations within a predicted structure of the hNav1.7 channel are shown in Figure 1A. To assess the biophysical properties of the PEM hNav1.7 mutant channels, the voltage-dependence of activation and fast inactivation was measured for the I848T and L858F mutants and compared to wild-type (WT) channels. WT hNav1.7 and the two mutant channels I848T and L858F were transiently co-expressed with the β1 and β2 Nav channel subunits in HEK293T cells (Figures 1B–D).

Previous studies indicated that related derivative channels exhibit a hyperpolarized shift in the voltage-dependence of activation, making mutant channels easier to open in response to small depolarizations. The voltage dependence of activation was assessed using a series of depolarizing voltage steps from a holding potential (*V*_h_) of −80 mV, where most hNav1.7 channels remain in the closed-resting state. Mutant channels activated at potentials 10–12 mV more negative than those required for WT channels (Figure 1E). The midpoint of activation (*V*_1/2_), estimated by fitting the data with a Boltzmann function, was significantly more negative for I848T currents (−34.6 ± 0.45 mV) and L858F currents (−36.6 ± 0.32 mV) than for WT currents (−21.2 ± 0.31 mV) (*p* < 0.0001, one-way ANOVA, Tukey’s multiple comparisons test; Figure 1E and Table 1). Similar peak current amplitudes were measured in cells expressing WT (−1,028 ± 163 pA at −10 mV; −36.71 ± 6 pA/pF), I848T (−1,235 ± 294 pA at −19 mV; −39.9 ± 9.5 pA/pF), and L858F (−1,607 ± 267 pA at −25 mV; −45.9 ± 5.9 pA/pF) channels. However, peak current amplitudes for the mutant channels occurred at more hyperpolarized potentials, consistent with the leftward shift in their activation curves.

**Table 1:** Voltage-dependent activation and steady-state fast inactivation parameters of wild-type and mutant hNav1.7 channels in the presence and absence of mepyramine.

				Fast inactivation			
	Activation			20 ms pre-pulse		200 ms pre-pulse	
	*n*	*V*_1/2_ (mV) CI (mV)	Slope *k* CI	*n*	*V*_1/2_ (mV) CI (mV)	*n*	*V*_1/2_ (mV) CI (mV)
WT							
CTL	11	−21.18 (−24.50 to −19.43)	3.91 (3.05 to 4.92)	11	−45.28 (−46.53 to −44.11)	7	−53.56 (−57.61 to −50.54)
MEPY	6	−21.48 (−23.63 to −19.31)	4.46 (3.54 to 5.3)	6	−44.09 (−45.71 to −42.58)	6	−67.15 (−69.67 to −72.89)
I848T							
CTL	7	−34.61 (−35.16 to −32.00)	3.42 (3.25 to 4.94)	6	−44.87 (−44.89 to −43.28)	5	−52.32 (−59.65 to −49.90)
MEPY	7	−32.26 (−32.47 to −30.05)	4.38 (3.39 to 5.58)	6	−46.25 (−47.99 to −44.65)	5	−62.91 (−74.3 to −60.54)
L858F							
CTL	9	−36.59 (−38.76 to −34.51)	3.91 (3.72 to 6.40)	8	−46.54 (−47.49 to −45.62)	4	−54.63 (−57.56 to −51.54)
MEPY	9	−37.30 (−39.01 to −35.60)	4.60 (3.25 to 6.43)	8	−47.04 (−48.59 to −45.57)	5	−67.20 (−73.90 to −64.80)
L1267V							
CTL	8	−20.02 (−20.87 to −18.79)	3.90 (3.35 to 4.50)	8	−42.40 (−44 0.02 to −37.91)	6	−51.52 (−56.78 to −49.02)
MEPY	8	−21.04 (−22.14 to −20.79)	4.73 (3.84 to 5.74)	6	−43.00 (−45.81 to −40.55)	6	−61.12 (−64.13 to −57.89)

Values are given as mean and upper and lower 95% confidence intervals (CI) are presented in brackets. CTL, control; MEPY, mepyramine.

Determination of fast inactivation properties using conditioning depolarizing pulses ranging from −80 to +10 mV revealed no significant difference between WT and I848T and L858F mutants (Table 1). Similarly, the voltage dependence of slow inactivation for I848T (*V*_1/2_ = −68.8 ± 4 mV, *p* = 0.919) and L858F (*V*_1/2_ = −67.25 ± 3.5 mV, *p* = 0.79) did not significantly differ from WT channels (*V*_1/2_ = −70.8 ± 3 mV) (Table 1). Thus, PEM mutations clustered in domain II of hNav1.7 cause a hyperpolarizing shift in the activation voltage, consistent with previous reports (20).

**Figure 2:**
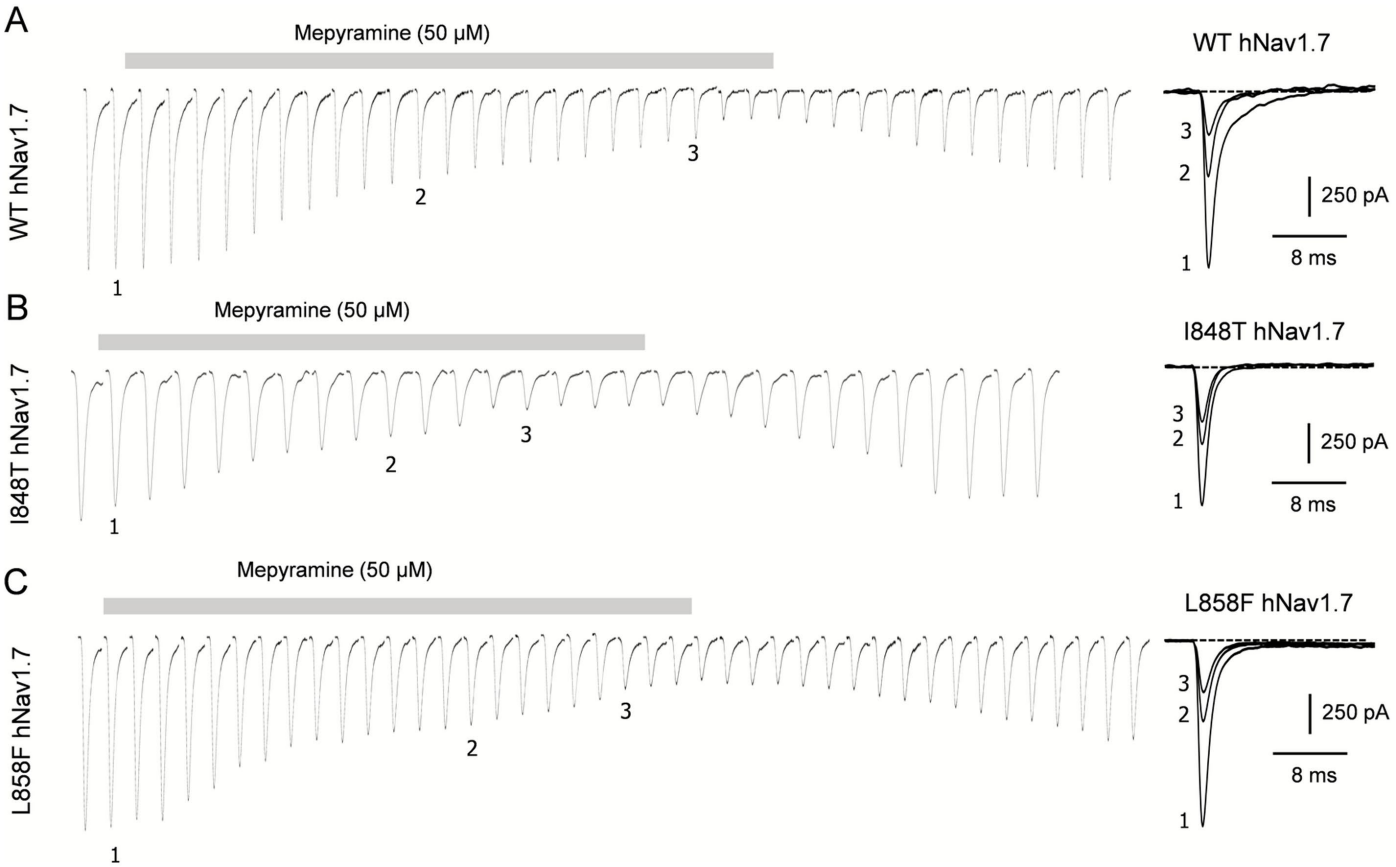
Time-dependent inhibition of I848T and L858F mutant channels by mepyramine. Concatenated recordings of wild-type **(A)**, I848T **(B)** and L858F **(C)** hNav1.7 channel currents exposed to 50 μM mepyramine. The currents were elicited by a 25-ms depolarizing pulse to 0 mV from a holding potential of −80 mV. The right insets show the development of current inhibition at the indicated time points. An inter-sweep interval of 7 s was applied to allow Nav channel recovery from inactivation. The inhibition is approximately 80% reversible within 15–20 min.

**Figure 3:**
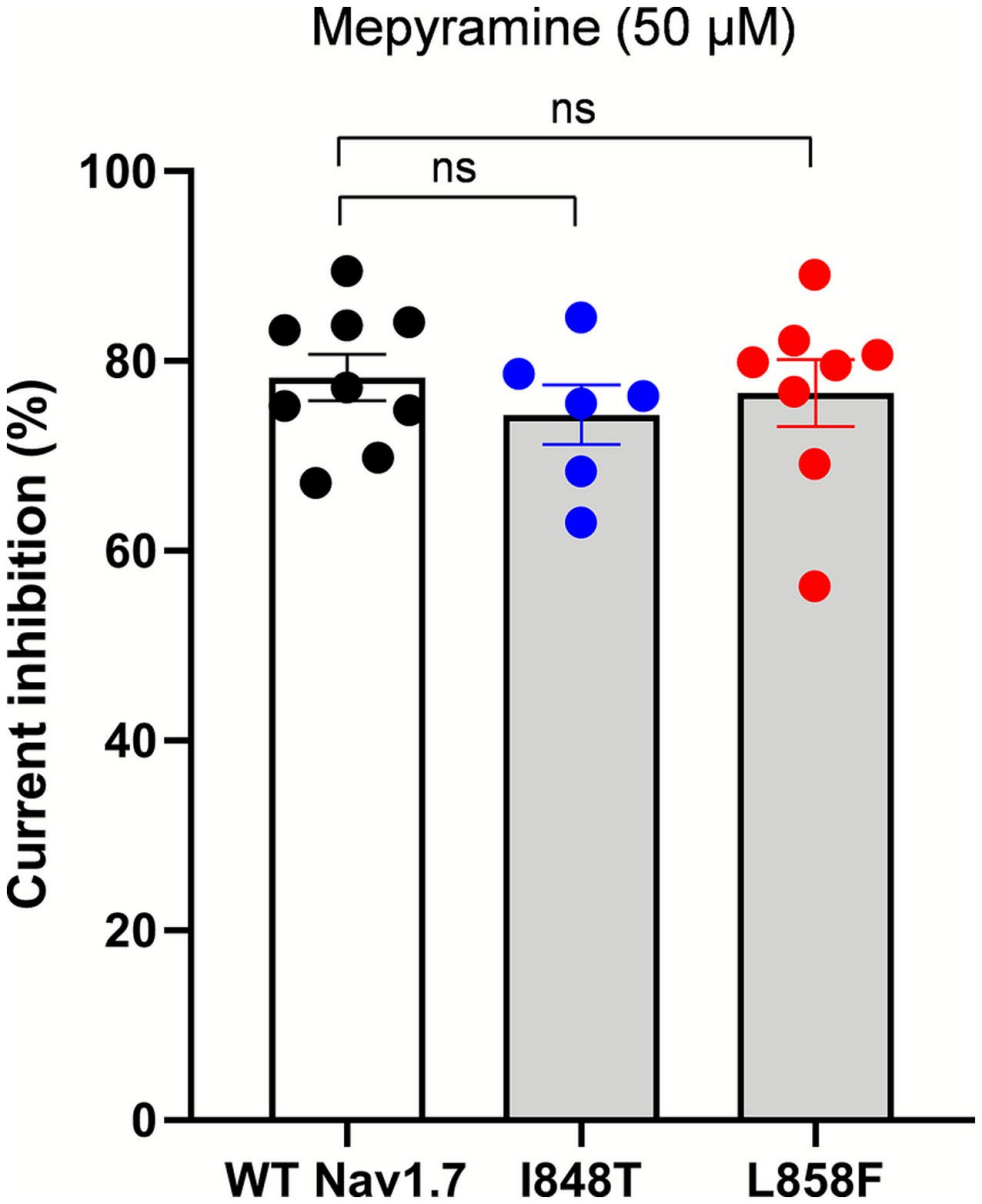
Mepyramine is equally effective in inhibiting the I848T and L858F mutant channels as it is in inhibiting wild-type channels. Normalized peak Nav1.7 current inhibition of wild-type (black circles), I848T (blue circles) and L858F (red circles) hNav1.7 currents by 50 μM mepyramine. Mepyramine was applied for at least 60 s to reach steady state block. Data are expressed as means ± SEM. Statistical analysis was performed using one-way ANOVA, Tukey’s multiple comparisons test. WT vs. I848T, *p* = 0.764; WT vs. L858F, *p* = 0.971.

The effects of mepyramine on mutant channels were assessed using a submaximal concentration of 50 μM (hNav1.7 IC_50_: 15.3 ± 0.1 μM) (35), which enabled the recording of inhibited currents while maintaining sufficient amplitude to analyze their biophysical properties. Mepyramine produced a graded inhibition of WT, I848T, and L858F hNav1.7 channels, with inhibition reversing slowly and incompletely (≈80%) during wash-out (Figure 2). The fractional block induced by mepyramine was calculated by dividing the peak amplitude of INav1.7 in the control condition by the INav1.7 amplitude in the presence of mepyramine. Mepyramine inhibited I848T and L858F by 73.5 ± 3.6% and 76.7 ± 3.5%, respectively, which were not significantly different from the 78.3 ± 2.5% inhibition observed in WT hNav1.7 channels (Figure 3).

**Figure 4:**
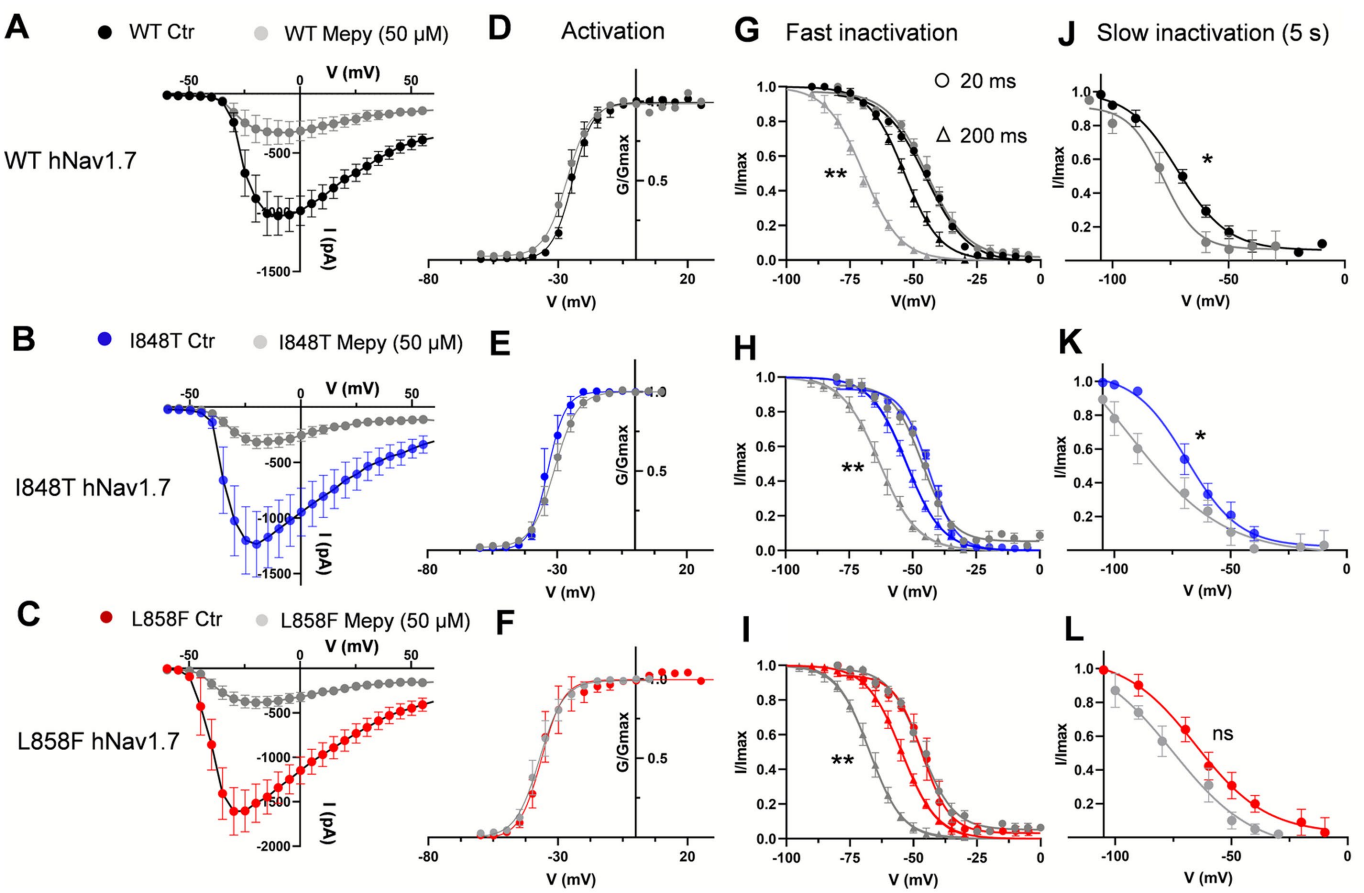
Mepyramine inhibits the I848T and L858F mutant channels by promoting inactivation. **(A–C)** Current–voltage relationships for wild-type (WT) **(A)**, I848T **(B)** and L858F **(C)** hNav1.7 currents, both before and after treatment with 50 μM mepyramine (grey symbols). Error bars indicate mean ± SEM. WT, *n* = 6–11; I848T, *n* = 7; L858F, *n* = 9. **(D–F)** Boltzmann fits of voltage-dependent activation of WT **(D)**, I848T **(E)** and L858F **(F)** hNav1.7, before and upon treatment with 50 μM mepyramine (grey circles). **(G–I)** Boltzmann fits of voltage-dependent fast inactivation of WT **(G)**, I848T **(H)** and L858F **(I)** hNav1.7, before and upon treatment with 50 μM mepyramine (grey symbols). Circles: 20 ms depolarization pre-pulse protocol; triangles: 200 ms depolarization pre-pulse protocol. ^**^p < 0.01. *V*_1/2_ fast inactivation (200 ms protocol): WT vs. WT MEPY, *p* = 0.004; I848T vs. I848T MEPY, *p* = 0.008; L858F MEPY, *p* = 0.006. One-way ANOVA, Sidak’s multiple comparisons test. **(J–L)** Boltzmann fits of voltage-dependent slow inactivation of WT **(J)**, I848T **(K)** and L858F **(L)** hNav1.7, before and upon treatment with 50 μM mepyramine (grey circles). ^*^p < 0.05; ns, not significant. *V*_1/2_ slow inactivation: WT vs. WT MEPY, *p* = 0.044; I848T vs. I848T MEPY, *p* = 0.032; L858F vs. L858F MEPY, *p* = 0.057. One-way ANOVA, Sidak’s multiple comparisons test.

Mepyramine had no significant effect on the voltage dependence of activation for either I848T (*V*_1/2_ = −32.26 mV ± 0.5 with mepyramine vs. −34.6 ± 0.45 mV in control) or L858F (*V*_1/2_ = −37.30 ± 0.41 mV with mepyramine vs. −36.6 ± 0.32 mV in control) (Figures 4A–F). However, its effect on the development of fast inactivation differed. While no significant differences were observed between I848T and L858F compared to WT in fast inactivation evoked with short depolarizing voltage pre-pulses, mepyramine caused a leftward shift of 10–13 mV in steady-state fast inactivation (Figures 4G–I and Table 1). Consistently, mepyramine induced a leftward shift in the slow inactivation parameters of WT, I848T, and L858F channels by 8–14 mV (Figures 4J–L and Table 2), indicating a preference for the slower, deeper inactivated states. These findings highlight that while mepyramine minimally impacts activation properties, it strongly modulates both fast and slow inactivation, particularly under conditions that favor deeper inactivation states. By this means, mepyramine can counteract the pathological effects of gain-of-function DII mutations affecting activation.

**Table 2:** Steady-state slow inactivation parameters of wild-type and mutant hNav1.7 channels in the presence and absence of mepyramine.

	*n*	*V*_1/2_ (mV) CI (mV)
WT		
CTL	5	−70.75 (−77.15 to −68.20)
MEPY	5	−78.20 (−83.5 to −70.98)
I848T		
CTL	5	−68.82 (−72.10 to −66.22)
MEPY	4	−82.55 (−87.50 to −74.00)
L858F		
CTL	4	−67.25 (−71.64 to −61.12)
MEPY	4	−75.20 (−82.01 to −73.60)
L1267V		
CTL	6	−62.60 (−69.87 to −56.85)
MEPY	6	−76.25 (−79.14 to −62.45)

Values are given as mean and upper and lower 95% confidence intervals (CI) are presented in brackets. CTL, control; MEPY, mepyramine.

### Mepyramine inhibits the PEM L1267V Nav1.7 variant

**Figure 5:**
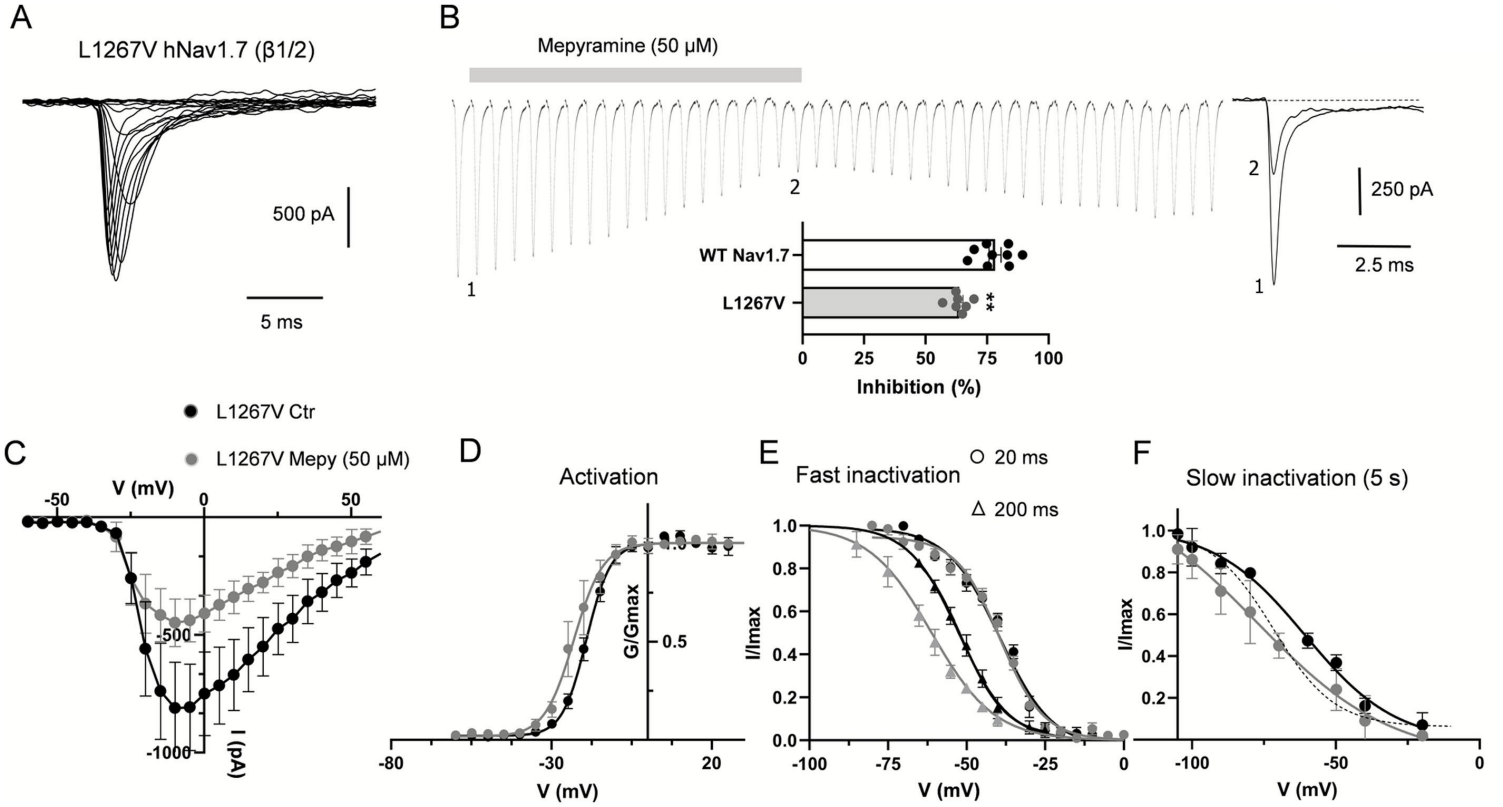
Mepyramine counteracts the gain-of-function effect of the L1267V mutation. **(A)** Current traces recorded from HEK293 cells co-expressing L1267V mutant channels with β1/β2-subunits. Cells were held at −80 mV and currents were elicited with 25 ms-test pulses to potentials ranging from −60 to 60 mV. For the sake of clarity not all traces are shown. **(B)** Concatenated recordings of L1267V channel currents exposed to 50 μM mepyramine. The currents were elicited by a 25 ms depolarizing pulse to 0 mV from a holding potential of −80 mV. Right insets show recordings at the time points indicated. Inter-sweep period was 7 s to allow recovery of Nav channels from inactivation. The inhibition is partially reversible within 15–20 min. Bottom inset: percentage inhibition of WT and L1267V peak currents by mepyramine. Statistical analysis was performed using one-way ANOVA, Tukey’s multiple comparisons test. ^**^p < 0.01. WT vs. L1267V, *p* = 0.0043. **(C)** Current–voltage relationships for L1267V hNav1.7 currents before and after treatment with 50 μM mepyramine (grey circles). Error bars indicate mean ± SEM (*n* = 8). **(D–F)** Boltzmann fits of voltage-dependent activation **(D)**, fast inactivation (circles, 20 ms depolarization pre-pulse protocol; triangles, 200 ms depolarization pre-pulse protocol) **(E)** and slow inactivation (5 s depolarization pre-pulse protocol) **(F)** for L1267V currents before and after treatment with 50 μM mepyramine (grey symbols). **(F)** The dash line depicts the slow inactivation Boltzmann curve for WT channels in the absence of mepyramine.

We investigated the missense mutation (c.3799C>G, L1267V) in the third domain of the Nav1.7 *α*-subunit (see Figure 1A), which we are reporting here for the first time in a pediatric case. This mutation has previously been linked to adult-onset small fiber neuropathy (40). The proband, a 9-year-old female (patient 3, Table 3), experienced bilateral episodes of burning pain in her hands and feet. During these attacks, her feet and hands became warm and red. The pain episodes were triggered by activity or exposure to heat. Characterization of the channel’s biophysical properties revealed that the mutation did not significantly affect the *V*_1/2_ for activation or fast inactivation (Figures 5A,C,D,E and Table 1), nor the peak current (−37.2 ± 7.6 pA/pF vs. −33.2 ± 5.2/pF pA at −10 mV) compared to WT. In contrast, the biophysical signature of the L1267V mutation showed a depolarizing shift in the voltage-dependence of slow inactivation compared to WT (from −70.8 mV ± 3 in WT to −62.60 ± 3.5 mV) (Figure 5F and Table 2), predicting reduced channel inactivation under conditions of sustained depolarization.

**Table 3:** Clinical characteristics of patients and pain scores at baseline and after 3 months of mepyramine treatment.

Patient	Gender	Age (y)	*SCNA9* mutation	NPRS score (0–10)		MCID reduction (%)	Frequency of painful crisis	Mean duration of crisis (min)	
				Baseline	MEPY			Baseline	MEPY
1*	M	40	I848T	9	4	Much relief (55)	Unchanged	60	30
2*	M	16	I848T	9	5	Moderate relief (44)	Unchanged	60	30
3	F	9	L1267V	7	1	Much relief (85)	Reduced	30	15
4	M	4	Idiopathic	8	2	Much relief (75)	Reduced	60	15
5*	F	45	Idiopathic	9	2	Much relief (77)	Reduced	120	30
6	F	30	Idiopathic	9	1	Much relief (88)	Reduced	60	0
7*	M	13	Idiopathic	8	3	Much relief (62)	Unchanged	90	20

NPRS, Numeric Pain Rating Scale; MCID, minimally clinically important difference. Idiopathic PEM describes erythromelalgia in which no genetic, secondary, or systemic cause can be identified despite appropriate evaluation. (*) Placed next to the patient number denotes a documented family history of PEM.

Interestingly, mepyramine inhibited L1267V (Figures 5B,C) associated with a shift in the slow inactivation curve, partly reversing the biophysical effects of the L1267V mutation on the channel (Table 2 and Figure 5F). As a result, mepyramine inhibited the L1267V-generated current by 63.3 ± 1.7%, which is approximately 15% less than the inhibition observed for WT channels (78.3 ± 2.5%) (Figure 5B) Thus, mepyramine exerts a normalizing, albeit partial, effect on the pathological gating properties of L1267V.

Collectively, these results indicate that mepyramine remains effective in inhibiting gain-of-function PEM Nav1.7 channels, whether they exhibit enhanced activation or altered channel inactivation.

### Evaluating the effectiveness of mepyramine cream in PEM patients

We conducted an evaluation of the effectiveness and safety of high-dose topical mepyramine in PEM patients. Data from seven mepyramine cream-treated patients with PEM were analyzed in the study. This study population was made up of 3 females and 4 males and included 4 subjects ≤18 years old. The patients referred to our joint pain and dermatology consultation were those with PEM that persisted despite classic analgesic treatments. The demographic and clinical characteristics of the patients are shown in Table 3. None of the patients had organ failure. Of the patients included, all completed a 3-month treatment course.

### Effectiveness of mepyramine cream in PEM patients with the I848T mutation

Initially, we evaluated the effects of mepyramine cream in two PEM patients carrying the I848T missense mutation in the *SCN9A* gene. These patients (patients 1 and 2, Table 3) were male, aged 16 and 40, with symptoms affecting the feet and/or hands, characterized by intense, burning pain in the affected extremities, severe redness, and increased skin temperature, all of which were episodic in nature. There was no history of foot ulceration or autonomic symptoms. The patients experienced at least 6 pain attacks per day, with a mean NPRS score of 9/10, despite the use of oral analgesics, including morphine, fentanyl, ketamine, methadone, buprenorphine, and mexiletine (Tables 3, 4). At the time of the therapeutic trial with the cream, patients were taking both methadone (80 mg/day) and mexiletine (835 mg/day), yet these analgesics provided no significant relief. The patients were treated for 3 months with the add-on cream containing 20% mepyramine, applied during each pain attack.

**Table 4:** Previous oral and topical treatments with limited or no therapeutic benefits.

Patient	Trials of oral medications
1	Morphine, fentanyl, ketamine, methadone, hydromorphone, mirtazapine, rufinamide, mianserine, buprenorphine, mexiletine, ranexa, neurontin, laroxyl, zonisamide, jakavi, clonidine, topical amitriptyline, topical lidocaine, topical NSAID
2	Morphine, fentanyl, ketamine, methadone, mexiletine, jakavi, neurontin, laroxyl, zonisamide, topical amitriptyline, topical lidocaine, topical NSAID
3	Morphine, fentanyl, methadone, mexiletine, neurontin, laroxyl, zonisamide
4	Morphine, methadone, rivotril, laroxyl, zonisamide, aspirin, topical amitriptyline
5	Lyrica, laroxyl, nifedipine, neurontin, paracetamol, topical lidocaine, topical NSAID
6	Aspirine, tramadol, morphine, topical NSAID
7	Laroxyl, tramadol

After applying the mepyramine cream for 5 min, both patients reported a decrease in mean pain intensity, from severe (NPRS score: 9/10) at baseline to moderate (NPRS score: 4.5/10) during pain crises. The minimally clinically important difference (MCID), defined as the percentage change in NPRS reduction, indicated a ≈ 50% reduction in pain. While the frequency of the attacks remained unchanged (averaging 6 attacks per day), the duration of each attack was reduced from 1 h before treatment to 30 min during the third month of treatment.

### Effectiveness of mepyramine cream in a patient with the L1267V PEM mutation

The patient (female, patient 3, Table 3) was 9 years old at the time of examination and presented with symptoms affecting both the feet and hands, characterized by severe redness and burning pain in the affected extremities. There was no history of foot ulceration or autonomic symptoms. The patient was experiencing at least 3 pain attacks per day, with a mean NPRS score of 7/10, despite using oral analgesics, including morphine, fentanyl, methadone, mexiletine, neurontin, laroxyl, and zonisamide (Table 4). At the time of the study, the patient was receiving fentanyl (25 μg/h), mexiletine (501 mg/day), and rufinamide (600 mg/day) but experienced no relief. The patient was treated for 3 months with the add-on cream containing 20% mepyramine, applied during each pain attack.

**Figure 6:**
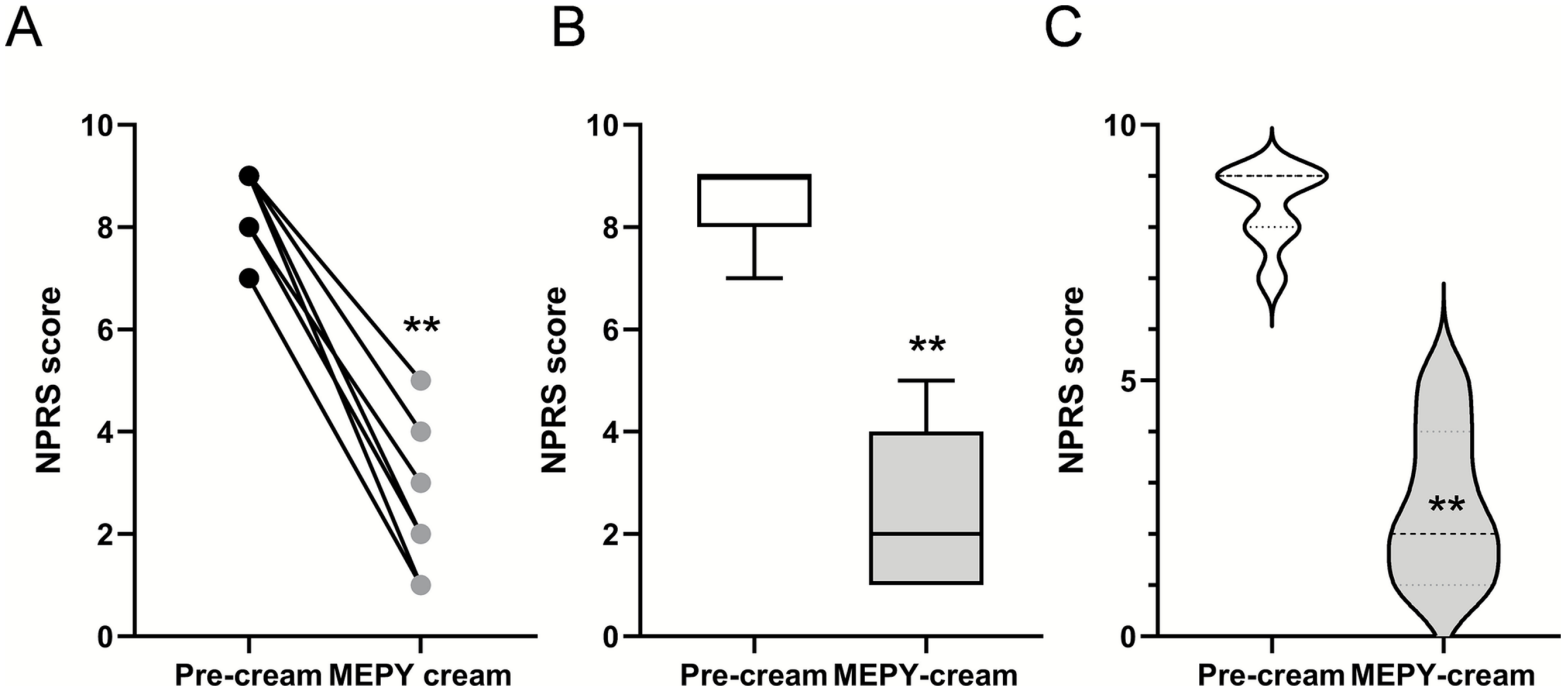
Localized 20% mepyramine application relieves burning pain in PEM patients. **(A)** Seven patients with PEM were treated with a 20% mepyramine hydrochloride cream, applied to each painful area during crises. Pain was assessed during attacks both without treatment and with mepyramine treatment using NPRS. Circles represent individual patient scores, and each line corresponds to one patient. **(B)** Box and whisker plots to show the distribution of NPRS scores in patients before and after mepyramine application. **(C)** Violin plots compare the density of NPRS scores in patients before and after mepyramine application. Statistical analysis was conducted using the Wilcoxon matched-pairs signed-rank test. ^**^p < 0.01.

**Figure 7:**
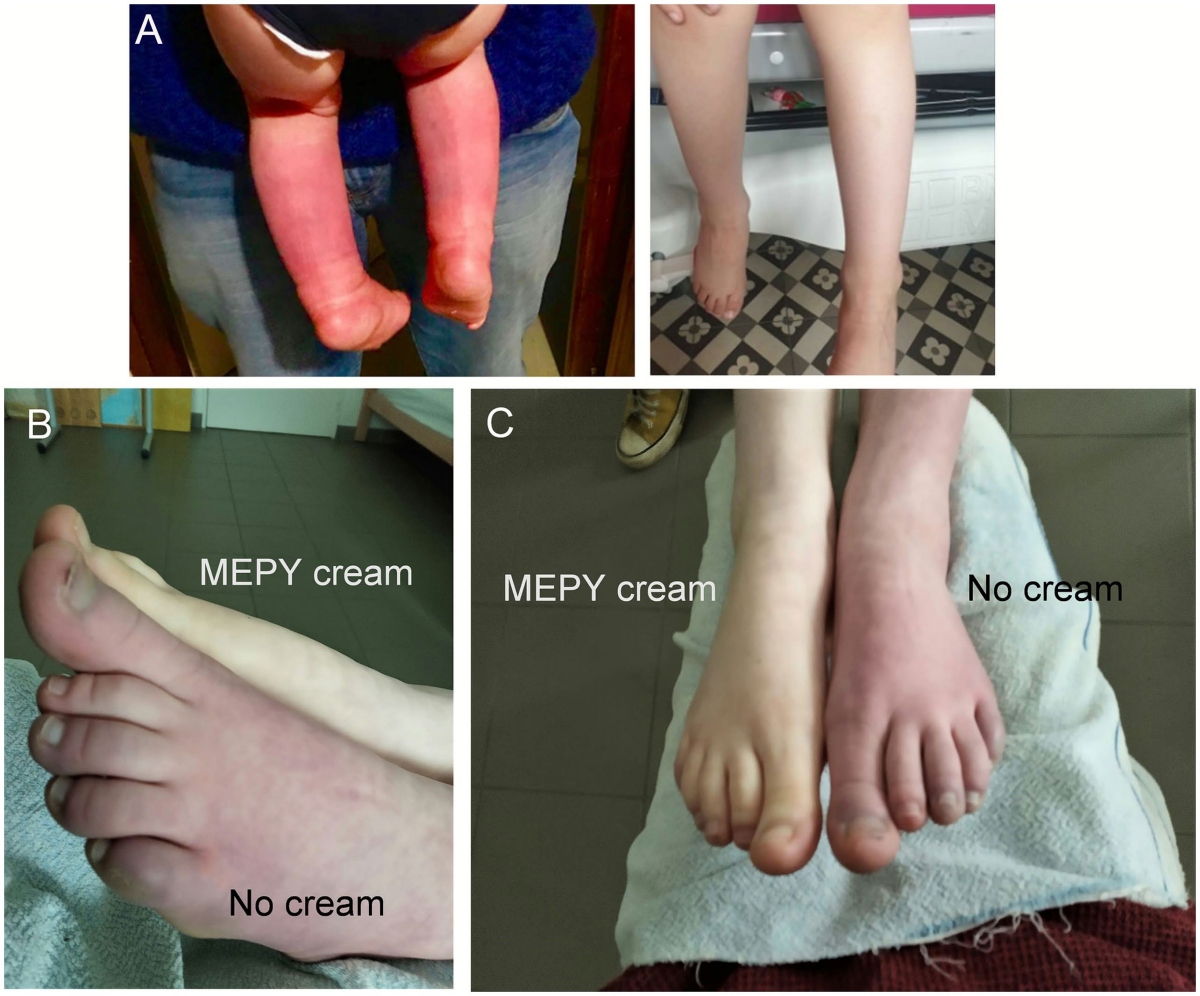
Mepyramine cream effectively reduces redness and flushing during crises. **(A)** Photographs of a 4-year-old Caucasian boy (patient 4, Table 3) with a several-month history of severe burning pain, redness, and swelling in both lower legs and feet (left image, age 4). The image on the right illustrates a reduction in erythema and painful flare-ups following treatment with topical mepyramine (age 5). **(B,C)** Phenotypic appearance of PEM during a crisis in a sporadic case with the L1267V mutation (patient 3, Table 3). Side view of the lower limbs **(B)** and front view **(C)**. The untreated left foot appears bright red, painful, and hot. Ten minutes after the local application of the mepyramine cream on the right foot, the patient experienced significant relief, with a marked reduction in pain intensity and visible resorption of skin redness. The quality of life for the patient and family has substantially improved. Images provided courtesy of the patient’s parents. The patient or their legal representatives were informed of this article’s contents and provided consent for publication.

The patient reported a rapid decrease in NPRS score from 7/10 at baseline to 1/10, along with the complete disappearance of erythema and redness following the topical application of mepyramine (Figures 6, 7B,C and Table 3). The MCID indicated much relief, with an 85% reduction in pain. Noticeable relief was observed within 5–10 min of applying and rubbing the mepyramine cream and lasted for at least 3–4 h. The frequency of the attacks was also drastically reduced, and when they did occur, the duration was shortened to 10 min after mepyramine treatment compared to 30 min at baseline. No school absenteeism was observed during treatment, and the 9-year-old girl was able to resume cross-country activities.

### Effectiveness of mepyramine cream in patients diagnosed with idiopathic PEM

Four patients [2 adults, aged 45 (F) and 30 (F), and 2 children, aged 4 (M) and 13 (M)] diagnosed with primary erythromelalgia (PEM) affecting the feet or both feet and hands were included in the case series (Table 3, patients 4 to 7) and treated with mepyramine cream for 3 months (Figures 6, 7A). The condition was considered idiopathic, as no underlying etiological mechanisms have yet been identified. These patients experienced episodic or intermittent crises, with episodes of painful, red-hot feet and/or hands. They were having at least 3 pain attacks per day, with a mean NPRS score of 8.5/10. Short trials of oral and topical medications were attempted (Table 4), but they proved ineffective. Specifically, at the time of the study, patient 4 was taking methadone (15 mg/day), zonisamide (50 mg/day), amitriptyline (15 mg/day), and aspirin (75 mg/day). Patient 5 was taking only paracetamol. Patient 6 was on amitriptyline (50 mg/day), whereas patient 7 was taking amitriptyline (25 mg/day) in combination with tramadol (150 mg/day). None of these medications had any significant effect on the intensity of the erythromelalgic episodes. Following mepyramine treatment, the patients reported a decrease in pain intensity from severe (NPRS score: 8.5/10) at baseline to mild (NPRS score: 2/10) (Table 3). The MCID demonstrated significant pain relief, with a remarkable 76% reduction in pain. Relief was rapid, with noticeable improvement within just 5–10 min of applying the cream. The frequency of attacks was significantly reduced for most patients, and the average duration of attacks markedly decreased from 90 min under baseline conditions to 17 min by the third month of mepyramine treatment. Notably, pain attacks were eliminated in one patient (patient 6, Table 3). Additionally, all these patients exhibited a significant resolution of erythema and redness following the topical application of mepyramine (Figure 7A). No AEs, such as skin irritation, nor any of the common systemic AEs associated with oral mepyramine use (sedation, headache, dizziness, somnolence/insomnia), were reported following the application of the cream by the patients in our study. Additionally, no changes in touch sensitivity or proprioception were reported.

## Discussion

Our findings demonstrate that mepyramine effectively inhibits the gain-of-function variants of hNav1.7 associated with PEM and that all patients with PEM respond positively to topical mepyramine treatment. Consequently, our study provides initial evidence suggesting that mepyramine cream holds promise as a potential therapeutic option for managing PEM.

In the absence of consensus or established guidelines for the treatment of PEM, the therapeutic approach to this challenging condition has largely relied on trial and error. Treatments for PEM aim to alleviate pain, reduce inflammation, and improve the patient’s quality of life. Pharmacological interventions often require customization based on individual patient responses, with a combination of medications frequently needed to achieve optimal outcomes (32). Moreover, PEM is notably resistant to many treatment options, further complicating its management (41).

Since PEM is often associated with mutations in the *SCN9A* gene (11), which cause hyperactivity of the Nav1.7 sodium channel, many treatments focus on modulating these specific mechanisms. Our data demonstrates that mepyramine effectively inhibits Nav1.7 channels with various PEM-associated mutations, including I848T, L858F and L1267V. Both I848T and L858F mutations caused a significant hyperpolarizing shift (~10–12 mV) in the voltage-dependence of activation compared to WT Nav1.7. These findings align with previous reports that domain II-associated mutations often shift activation parameters without affecting inactivation parameters (21, 40). This alteration lowers the activation threshold, making mutant channels more prone to opening in response to small depolarizations. On the contrary, the L1267V mutation had no effect on activation or fast inactivation properties but affected slow inactivation. This alteration likely enhances persistent channel activity, contributing to heightened neuronal excitability and pain crises (42, 43).

Mepyramine preferentially targets inactivation states induced by prolonged conditioning steps in both WT and mutant channels. By stabilizing Nav1.7 channels in the nonconductive inactivated state, mepyramine mitigates the hyperactive state caused by the I848T and L858F mutations. Additionally, it counteracts the pathological effects of the L1267V mutation. Thus, mutations enhancing open-state probability or impairing inactivation did alter the availability of mepyramine-bound conformations. This is particularly important because PEM mutations have been shown to reduce the channel’s affinity for local anesthetics, such as lidocaine and mexiletine, which may explain the refractoriness to these treatments (42, 44–46). Collectively, these findings indicate that mepyramine efficiently inhibits Nav1.7 activity regardless of whether the mutation impacts activation or inactivation dynamics, underscoring its broad therapeutic potential against various Nav1.7 gain-of-function mutations. Additionally, mepyramine inhibits WT Nav1.7 as well as other sodium channels, including Nav1.8 and Nav1.9 (35). This broader inhibitory profile highlights the efficacy of mepyramine cream in alleviating pain and erythema in PEM patients without *SCN9A* mutations (e.g., patients 4–7) as well as in other neuropathic conditions (unpublished data). mepyramine’s ability to inhibit a variety of mutant Nav1.7 channels, as well as other sodium channels, suggests it could address diverse presentations of PEM, even without detailed genetic information.

The pathophysiology of erythromelalgia involves neuropathic and vascular elements, with excessive vasodilation driven by hyperexcitable sensory neurons (47). These neurons release neurotransmitters like substance P and CGRP, potent vasodilators that increase skin blood flow and cause redness and warmth. Mepyramine mitigates these effects by inhibiting excitatory sodium channels, reducing paroxysmal vasodilation, and targeting histamine pathways implicated in neurogenic inflammation, pain, and vascular permeability (37). Although antihistamines alone are rarely effective, occasional therapeutic benefits are reported (48, 49). Thus, mepyramine alleviates the symptoms of erythromelalgia through dual mechanisms: inhibition of pain-transmitting sodium channels to reduce neuronal hyperexcitability and suppression of local vascular inflammation by targeting histamine pathways, thereby mitigating excessive vasodilation and neurogenic inflammation.

Our exploratory, observational study involving seven patients with PEM affecting the extremities suggests that topical 20% mepyramine cream effectively alleviate both pain and erythema. The cohort was diverse in terms of age, gender, and symptomatology, including patients with identified mutations, those without *SCN9A* mutations, and others with unknown genetic etiology. Patients suffered from frequent and intense pain episodes (mean NPRS score of 8.5/10), which were debilitating and resistant to conventional pharmacological treatments, including opioids (e.g., morphine, methadone) and other oral or topical analgesics. Mepyramine cream demonstrated remarkable efficacy in reducing the intensity of PEM crises. Mean pain intensity decreased by 70%, with mean NPRS score dropping to a manageable level of 2.5/10. Additionally, the frequency and duration of episodes were markedly reduced, with complete remission observed in one patient. The results were sustained over a 3-month treatment period, and most patients continued using mepyramine cream after two years, indicating sustained tolerability and patient-perceived benefit for PEM symptom management. Importantly, except for patients 1 and 2, all other patients were able to taper and discontinue opioids and mexiletine, reducing exposure to their addictive and adverse effects. In addition, the use of a topical formulation minimizes systemic exposure, potentially reducing the risk of off-target effects while concentrating the therapeutic effect at the site of pain. Notably, no adverse side effects were reported during the two-year treatment period, further supporting its favorable safety profile.

While our patient cohort includes only seven individuals, it is critical to note that PEM is an ultra-rare disease, affecting approximately 1 in 1,000,000 individuals, with only 25 families identified across France. In this context, the inclusion of seven well-characterized patients represents a substantial proportion of the national PEM population. A limitation of this study is that the patient cohort, consisting of heavily pretreated individuals, may not be generalizable to all PEM patients. Multiple commercially available topical analgesic formulations, including amitriptyline, lidocaine, and NSAID-based creams (Table 4), were tested in the same patient cohort but failed to provide meaningful pain relief. The consistent lack of efficacy of these agents, which contrasted with the positive effect observed with mepyramine, provides a strong form of internal control and underscores both the specificity and therapeutic potential of mepyramine. At the initial stage of the study, we also prepared formulations containing low concentrations of mepyramine (2–5%), which similarly demonstrated no significant analgesic effect. These formulations can be considered an additional internal control, further supporting the dose-dependent efficacy observed with the 20% preparation. Furthermore, in a subset of patients, the topical mepyramine cream was applied unilaterally. In these cases, symptom relief was observed exclusively in the treated limb, whereas the contralateral, untreated limb remained painful. This side-specific response strongly suggests that the therapeutic effect is pharmacological in nature rather than attributable to a placebo effect.

Although mepyramine cream showed marked efficacy in reducing the intensity of PEM crises in our small cohort, its effectiveness must be evaluated in a larger PEM population. Translating promising results from small, well-characterized groups to broader patient populations can be challenging, even within the same disease. For example, Cao et al. (50) reported that a selective Nav1.7 blocker from Pfizer produced robust improvements in an initial study of patients with inherited erythromelalgia, yet these results did not extend to larger cohorts. This underscores the importance of validating our findings in a more diverse and expanded PEM patient population.

In conclusion, mepyramine may offer a potential therapeutic benefit for patients with this debilitating condition, effectively inhibiting Nav1.7 channels across multiple gain-of-function mutations. Further clinical studies are required to confirm mepyramine’s therapeutic potential and explore its role in precision medicine for Nav1.7- and other sodium channel–related disorders.

## Data Availability

The original contributions presented in the study are included in the article/supplementary material, further inquiries can be directed to the corresponding author.
